# SGTA regulates the cytosolic quality control of hydrophobic substrates

**DOI:** 10.1242/jcs.155648

**Published:** 2014-11-01

**Authors:** Lydia Wunderley, Pawel Leznicki, Aishwarya Payapilly, Stephen High

**Affiliations:** Faculty of Life Sciences, University of Manchester, Oxford Road, Manchester M13 9PT, UK

**Keywords:** Aggresomes, BAG6, Mislocalised membrane proteins, Polyubiquitylation, Protein degradation, Small glutamine-rich tetratricopeptide repeat-containing protein alpha

## Abstract

Hydrophobic amino acids are normally shielded from the cytosol and their exposure is often used as an indicator of protein misfolding to enable the chaperone-mediated recognition and quality control of aberrant polypeptides. Mislocalised membrane proteins (MLPs) represent a particular challenge to cellular quality control, and, in this study, membrane protein fragments have been exploited to study a specialised pathway that underlies the efficient detection and proteasomal degradation of MLPs. Our data show that the BAG6 complex and SGTA compete for cytosolic MLPs by recognition of their exposed hydrophobicity, and the data suggest that SGTA acts to maintain these substrates in a non-ubiquitylated state. Hence, SGTA might counter the actions of BAG6 to delay the ubiquitylation of specific precursors and thereby increase their opportunity for successful post-translational delivery to the endoplasmic reticulum. However, when SGTA is overexpressed, the normally efficient removal of aberrant MLPs is delayed, increasing their steady-state level and promoting aggregation. Our data suggest that SGTA regulates the cellular fate of a range of hydrophobic polypeptides should they become exposed to the cytosol.

## INTRODUCTION

Hydrophobic amino acids are typically buried within the tertiary structure of a protein, and their inappropriate exposure can be exploited by cellular quality control components as an indicator of misfolding (Buchberger et al., 2010). The contiguous stretches of hydrophobic amino acids that characterise integral membrane proteins provide a particularly extreme and aggregation-prone indicator of misfolding when such polypeptides mislocalise to the cytosol (Ast and Schuldiner, 2011; Leznicki et al., 2013; Rodrigo-Brenni and Hegde, 2012). For membrane proteins entering the eukaryotic secretory pathway, their signal recognition particle (SRP)-dependent delivery to the endoplasmic reticulum (ER) and subsequent co-translational integration by the Sec61 translocon effectively reduces the opportunity for any cytosolic exposure of their transmembrane domains. Hence, the first transmembrane domain to emerge from the ribosomes acts as an ER-targeting signal that is bound to and shielded by the SRP54 subunit (Cross et al., 2009), whereas any subsequent hydrophobic regions will be protected from the cytosol by a combination of the ribosome and ER translocon (Hou et al., 2012).

In practice, the efficiency of SRP-dependent targeting is influenced by various factors, including the hydrophobicity and location of the ER-targeting signal (Goder et al., 2000), and the process is also subject to regulation (Swanton and High, 2006). Furthermore, a number of membrane and secretory proteins are delivered to the ER by post-translational SRP-independent pathways that might also result in the mislocalisation of some precursors to the cytosol (Ast et al., 2013; Johnson et al., 2013; Rodrigo-Brenni and Hegde, 2012). Whatever their precise origin, it is clear that higher eukaryotes have a distinct quality control pathway that deals with mislocalised membrane and secretory proteins (MLPs) (Hessa et al., 2011; Kawahara et al., 2013; Leznicki and High, 2012; Rodrigo-Brenni and Hegde, 2012). Notably, key components that mediate MLP quality control also contribute to the ER-associated degradation (ERAD) of misfolded membrane proteins and the biogenesis of selected precursors destined for the ER (Johnson et al., 2013; Leznicki et al., 2010; Mariappan et al., 2010; Rodrigo-Brenni and Hegde, 2012).

The BAG6 complex and the small glutamine-rich tetratricopeptide repeat-containing protein α (SGTA) play a central role in dictating the fate of MLPs (Hessa et al., 2011; Leznicki and High, 2012) and promote the insertion of tail-anchored proteins into the ER membrane (Kawahara et al., 2013; Leznicki et al., 2010; Leznicki et al., 2013; Leznicki et al., 2011; Mariappan et al., 2010). In the latter case, BAG6 and SGTA act upstream of an ATP-dependent targeting factor, TRC40 (also known as ASNA1) (Chartron et al., 2012; Leznicki et al., 2010; Mariappan et al., 2010), which delivers precursors to the ER for post-translational membrane insertion and translocation (Favaloro et al., 2008; Johnson et al., 2012; Stefanovic and Hegde, 2007). During the cytosolic quality control of aberrant precursors, BAG6 and SGTA appear to act in tandem to control the polyubiquitylation status, and hence proteasomal degradation, of MLPs. Previous studies have suggested that BAG6 acts to promote the polyubiquitylation of hydrophobic substrates, including MLPs (Hessa et al., 2011; Minami et al., 2010), whereas SGTA antagonises this process by enabling substrate deubiquitylation (Leznicki and High, 2012). This functional overlap between SGTA and the BAG6 complex is mirrored by a specific physical interaction between an SGTA dimer and ubiquitin-like domains present on subunits of the BAG6 complex (Chartron et al., 2012; Leznicki et al., 2013; Simon et al., 2013; Xu et al., 2012). Furthermore, both components are also implicated in the ERAD of aberrant membrane proteins synthesised at the ER (Claessen and Ploegh, 2011; Wang et al., 2011; Xu et al., 2012; Xu et al., 2013). Hence, SGTA and the BAG6 complex appear to deal with a range of substrates that feature cytosolically exposed regions of hydrophobicity, including transmembrane regions that are not membrane embedded (Kawahara et al., 2013; Leznicki et al., 2013).

In this study, we have focused on the role of SGTA during the specific recognition and degradation of aberrant membrane protein precursors that expose a hydrophobic degron to the cytosol. To this end, we have used short N-terminal fragments of polytopic membrane proteins to create model MLPs, and we studied their fate following perturbations of SGTA and BAG6 in mammalian cells. One of these MLPs, a fragment of TASK1 (also known as KCNK3) with an opsin N-glycosylation tag (OPG–TASK_85_), is almost exclusively mislocalised to the cytosol and interacts with both BAG6 and SGTA through its hydrophobic transmembrane domain. Consistent with their suggested opposing actions, the steady-state level of OPG–TASK_85_ is specifically increased upon BAG6 knockdown but reduced upon SGTA knockdown. The overexpression of exogenous SGTA selectively stabilises MLP and ERAD substrates by delaying their normal proteasomal degradation. This non-physiological stabilisation of MLPs requires SGTA to maintain an intact BAG6-binding site, and our data suggest that SGTA can promote MLP deubiquitylation. In a physiological context, such substrate deubiquitylation most likely provides a potential rescue pathway that is employed by specific precursors such as tail-anchored membrane proteins. We conclude that the combined actions of SGTA and the BAG6 complex normally act to ensure the efficient removal of MLPs, thereby preventing their accumulation and potential aggregation and minimising potentially harmful perturbations of normal cellular proteostasis (Buchberger et al., 2010; Chakrabarti and Hegde, 2009; Hartl et al., 2011; Park et al., 2013), whilst at the same time promoting the correct maturation of selected hydrophobic precursor proteins destined for the ER.

## RESULTS

Previous studies have suggested that SGTA regulates the fate of aberrant membrane proteins that are localised in the cytosol as a consequence of both inefficient delivery to (Leznicki and High, 2012), and retrotranslocation from (Xu et al., 2012), the ER. Naturally occurring nonsense mutations sometimes generate very short hydrophobic fragments derived from complex membrane proteins including opsin (Jacobson et al., 1994), and we speculated that such polypeptides might be especially prone to cytosolic mislocalisation (see also Heymann and Subramaniam, 1997), thereby providing potential model substrates for studying the role of SGTA during the cytosolic quality control of MLPs.

### Exogenous SGTA increases the steady-state level of cytosolic MLPs

**Fig. 1.**
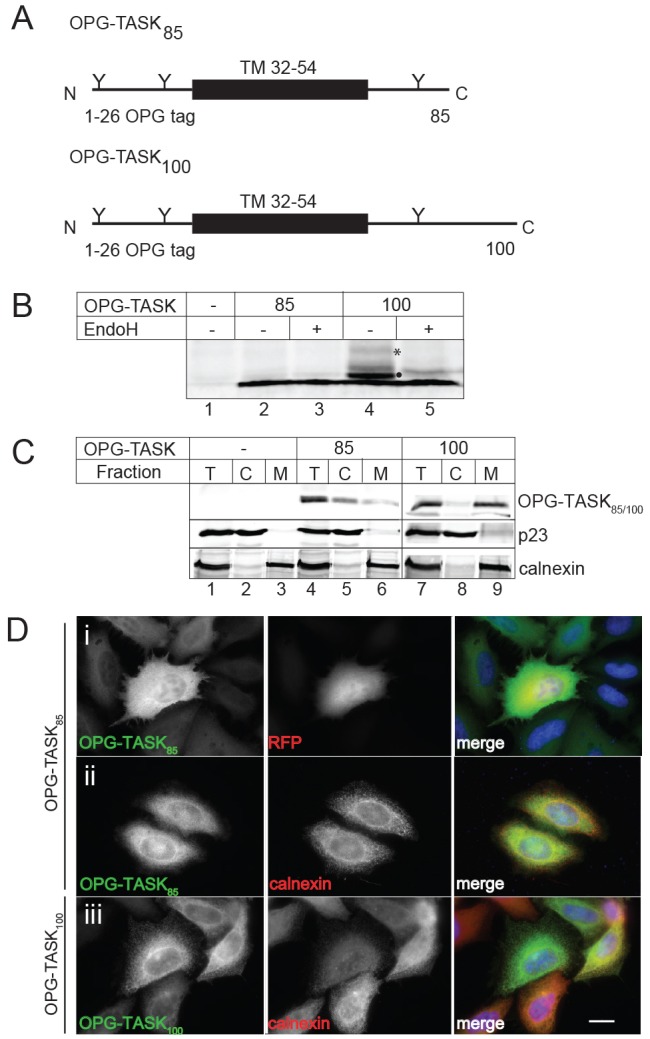
**OPG–TASK_85_ is a mislocalised protein.** (A) Schematics of the OPG–TASK_85_ and OPG–TASK_100_ polypeptides comprising the first 26 residues of bovine opsin followed by amino acids 2–60 or 2–75 of TASK-1. The opsin-derived N-glycosylation sites (Y) and TM1 of TASK-1 (TM 32–54) are indicated. (B) HeLaM cells were untransfected (lane 1) or transiently transfected with either OPG–TASK_85_ (lanes 2 and 3) or OPG–TASK_100_ (lanes 4 and 5), and total cell lysates were treated with endoglycosidase H (EndoH) where indicated (+) before analysis by western blotting using an anti-opsin monoclonal antibody. Singly (closed circle) and doubly (*) glycosylated forms of OPG–TASK_100_ are indicated. (C) Cells were prepared as for B and a portion was removed as a sample of the input (T), and the rest of the cells were lysed mechanically before separating the membrane (M) and cytosolic (C) fractions by ultracentrifugation. Equivalent amounts of the resulting samples were analysed by western blotting using antibodies against opsin to reveal the TASK-1-derived products and against p23 and calnexin as markers for the cytosolic and membrane fractions, respectively. (D) Cells were co-transfected with OPG–TASK_85_ and RFP (i), OPG–TASK_85_ (ii) or OPG–TASK_100_ (iii) as indicated, fixed and analysed for OPG–TASK (anti-opsin), RFP (direct visualisation) or calnexin (anti-calnexin) by fluorescence microscopy. Scale bar: 20 µm.

During a study of TASK-1 biogenesis, we found that a short N-terminal fragment, incorporating only the first of its four transmembrane spans, was efficiently inserted into ER-derived microsomes when synthesised using a cell-free system (Watson et al., 2013). By contrast, when the same TASK-1-derived fragment (hereafter referred to as OPG–TASK_85_, Fig. 1A) was expressed in HeLa cells we found no evidence for its membrane insertion. Hence, OPG–TASK_85_ is not detectably N-glycosylated at either N- or C-terminal reporters (Fig. 1B, cf. lanes 2 and 3). By contrast, increasing the chain length of the truncated TASK-1 derivative by only 15 residues, to create OPG–TASK_100_, resulted in clearly detectable levels of N-glycosylation (Fig. 1A, OPG–TASK_100_; Fig. 1B, cf. lanes 2–5). Subcellular fractionation (Fig. 1C, cf. lanes 5, 6, 8 and 9) and immunofluorescence microscopy (Fig. 1D) confirmed that most, if not all, of the shorter OPG–TASK_85_ chains were cytosolic, whereas a substantial proportion of the slightly longer OPG–TASK_100_ chains were associated with the ER membrane. The mislocalisation of OPG–TASK_85_ to the cytosol is most likely a consequence of a decreased window of opportunity for SRP to bind to the first transmembrane domain (TM1), the only ER-targeting signal present in the truncated TASK-1-derived fragment, before translation termination occurs *in vivo* (Goder et al., 2000), thereby precluding its efficient co-translational delivery to the ER (Cross et al., 2009). In this scenario, extending the C-terminus of the TASK-1-derived fragment by as little as fifteen residues would increase the likelihood of successful SRP-dependent co-translational targeting to the ER.

**Fig. 2.**
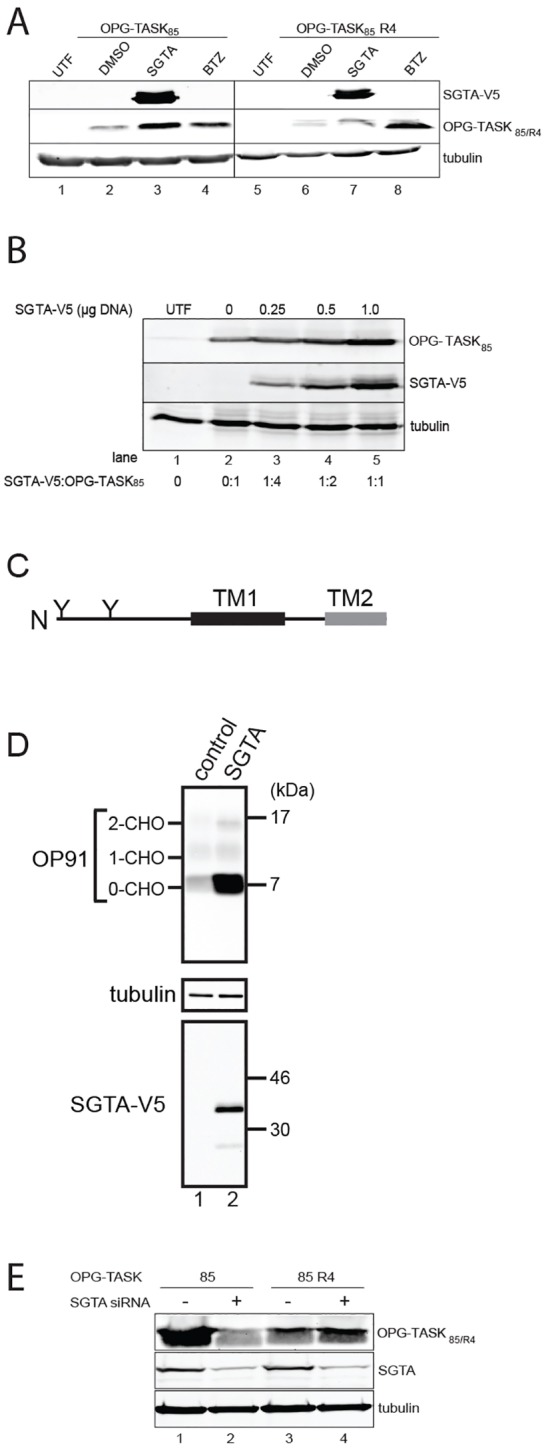
**Exogenous SGTA increases steady-state MLP levels.** (A) Cells were left untransfected and untreated (UTF, lanes 1 and 5) or were transfected with OPG–TASK_85_ (lanes 2–4) or OPG–TASK_85_ R4 (lanes 6–8). Samples were further treated with DMSO (lanes 2 and 6) or 10 nM bortezomib (BTZ, lanes 4 and 8) for 18 hours or co-transfected with SGTA–V5 (lanes 3 and 7) before total cell lysates were prepared and analysed by western blotting. Samples were probed for V5-tagged SGTA and OPG–TASK_85_ variants as indicated. Endogenous tubulin provided a loading control. (B) Cells were co-transfected with a fixed amount of OPG–TASK_85_ plus a combination of plasmids encoding SGTA–V5 and RFP, such that the total amount of DNA present was constant. Total cell lysates were analysed as for A. (C) Schematic of the OP91 polypeptide comprising the first 91 amino acids of bovine opsin, containing two sites for N-linked glycosylation (Y), the first hydrophobic transmembrane region (TM1) and part of the second (TM2). (D) HeLa cells were co-transfected with plasmids encoding OP91 and empty vector (control) or SGTA–V5. Total cell lysates were analysed at 24 hours after transfection as for A. (E) Cells were transfected with a control or SGTA-targeting siRNA duplex as indicated and then re-transfected with OPG–TASK_85_ (85) or OPG–TASK_85_ R4 (85 R4). After 24 hours, total cell lysates were analysed by western blotting as before except endogenous SGTA was detected.

The recognition of MLPs by key components implicated in their cytosolic quality control, namely the BAG6 complex and SGTA, appears to rely on exposed hydrophobic residues, including non-membrane-inserted transmembrane domains (Hessa et al., 2011; Leznicki and High, 2012). We therefore used OPG–TASK_85_ as a candidate MLP (Fig. 1) in order to test the role of cytosolically exposed hydrophobicity in directing proteins towards a specific pathway for degradation (cf. Leznicki and High, 2012). To this end, we generated OPG–TASK_85_ R4, an alternative version of OPG–TASK_85_ where the transmembrane region is strongly perturbed by the introduction of two pairs of arginine residues (supplementary material Fig. S1A,B; cf. Hessa et al., 2011). Overexpression has been used to study the role of several components implicated in protein quality control and degradation (cf. Christianson et al., 2012; Fleig et al., 2012; Guerriero and Brodsky, 2012; Liu et al., 2014), and we found that the coexpression of exogenous SGTA increased the steady-state level of OPG–TASK_85_ substantially (Fig. 2A, cf. lanes 2 and 3), consistent with our previous study (Leznicki and High, 2012). Furthermore, the steady-state level of OPG–TASK_85_ correlated with the amount of exogenous SGTA that was expressed, suggesting that it is the total amount of cellular SGTA that influences the level of OPG–TASK_85_ (Fig. 2B). By contrast, SGTA expression had little if any effect on OPG–TASK_85_ R4 (Fig. 2A, cf. lanes 6 and 7), although steady-state levels of both forms of OPG–TASK_85_ were increased upon treatment with the proteasome inhibitor bortezomib (Fig. 2A, cf. lanes 2, 4, 6 and 8). We conclude that although both TASK-1-derived fragments are substrates for proteasomal degradation, only the removal of the OPG–TASK_85_ substrate with an intact transmembrane region that acts as a ‘hydrophobic degron’ (cf. Minami et al., 2010) is affected by the perturbation of cellular SGTA levels.

*In vitro* analysis suggested that a short N-terminal segment of opsin might also be a substrate for SGTA-mediated quality control (Leznicki and High, 2012), and when this OP91 fragment (Fig. 2C) was transiently expressed in HeLa cells alone, the resulting products were only faintly visible by western blotting (Fig. 2D, lane 1). Treatment with bortezomib resulted in a substantial increase in OP91 levels, whereas inhibitors of lysosomal degradation had no effect, suggesting that OP91 is subject to proteasomal degradation (supplementary material Fig. S1C). Most strikingly, we also observed a large increase in the level of OP91 upon SGTA coexpression (approximately sevenfold enhancement; Fig. 2D; supplementary material Fig. S1D). It should be noted that both bortezomib treatment and SGTA coexpression selectively enhanced the levels of a discrete non-N-glycosylated OP91 species (OP91 0-CH0), consistent with the proposal that opsin-derived polypeptides located in the cytosol are selectively stabilised in both cases (Fig. 2D; supplementary material Fig. S1C,D). However, unlike bortezomib, the effects of SGTA coexpression were substrate specific, and the proteasomal degradation of an N-end rule substrate, Ub-R–GFP was unaffected (supplementary material Fig. S1E). Taken together, these data indicate that a substantial proportion of OP91 acts as an MLP that fails to reach the ER and defaults to the cytosol, where it acts as a substrate for BAG6- and SGTA-mediated quality control (cf. Hessa et al., 2011; Leznicki and High, 2012).

In order to further test the role of cellular SGTA in the quality control of MLPs, we reduced the level of the endogenous protein using an siRNA approach and studied the effect on steady-state OPG–TASK_85_. The loss of cellular SGTA resulted in a reduction in the amount of OPG–TASK_85_ (Fig. 2E, cf. lanes 1 and 2; supplementary material Fig. S1F), the opposite to the effect of SGTA overexpression (Fig. 2B). Likewise, a knockdown of SGTA also resulted in a loss of steady-state OP91, our second model MLP (supplementary material Fig. S1G), but had no effect on either OPG–TASK_85_ R4 (Fig. 2E, cf. lanes 3 and 4) or Ub-R–GFP (supplementary material Fig. S1H). Taken together, these data suggest that the absolute amount of cellular SGTA has a direct influence on the proteasomal degradation of MLPs. In contrast to OPG–TASK_85_, we found that a small fraction of OP91 is N-glycosylated (Fig. 2D; supplementary material Fig. S1C,D), indicative of some membrane integration, and this population showed a modest increase upon coexpression with SGTA (supplementary material Fig. S1D, see OP91 1-CHO and 2-CHO levels). We conclude that some fraction of newly synthesised OP91 is most likely a potential substrate for ERAD, a process in which both BAG6 and SGTA also participate (Liu et al., 2014; Wang et al., 2011; Xu et al., 2012), and we therefore employed OPG–TASK_85_ as our principal model MLP for further study.

### SGTA and BAG6 cooperate to regulate MLP degradation

**Fig. 3.**
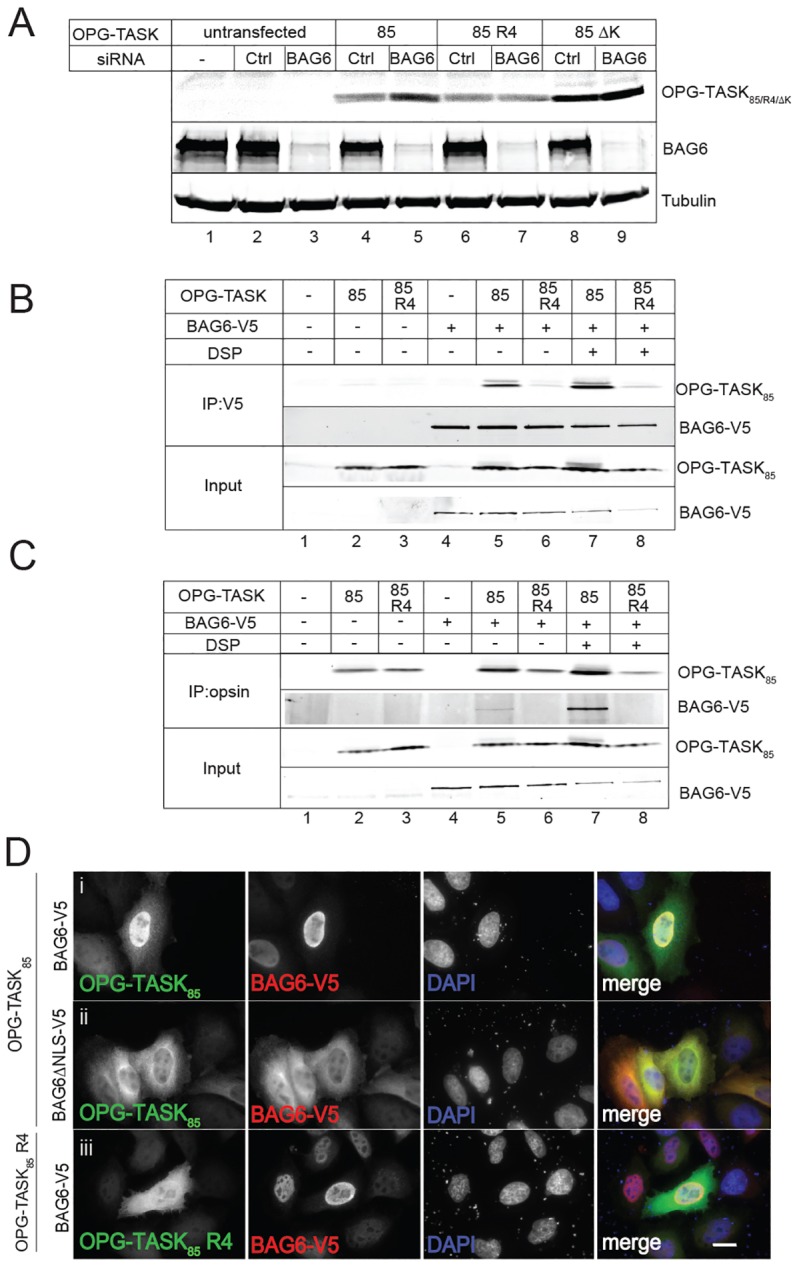
**OPG–TASK_85_ is a substrate for BAG6.** (A) HeLaM cells were left untransfected, transfected with an siRNA control (Ctrl) or a duplex targeting BAG6, as indicated, and then were either not further manipulated or were re-transfected with OPG–TASK_85_ (85), OPG–TASK_85_ R4 (85 R4) or a lysine-deficient OPG–TASK_85_ (85 ΔK). After 24 hours, total cell lysates were analysed by western blotting. (B,C) Cells were co-transfected with OPG–TASK_85_ or OPG–TASK_85_ R4 and BAG6–V5, as indicated, and 24 hours later, incubated with DSP (+) or solvent (DMSO) alone (−), quenched and lysed in buffer containing 0.5% DDM. BAG6–V5 (B) or OPG–TASK_85_ (C) were immunoprecipitated (IP) with anti-V5 or anti-opsin, respectively, and the material recovered was then analysed by western blotting using 10% of the input for comparison. (D) Cells were co-transfected with OPG–TASK_85_ (i,ii) or OPG–TASK_85_ R4 (iii) and BAG6–V5 (i,iii) or BAG6–V5 ΔNLS (ii), as indicated, and were processed as for Fig. 1D using antibodies recognising OPG–TASK (anti-opsin tag) and exogenous BAG6 (anti-V5 tag). Scale bar: 20 µm.

Our previous work suggested that SGTA influences the degradation of MLPs by antagonising the actions of the BAG6 complex (Leznicki and High, 2012), and we therefore next asked whether OPG–TASK_85_ is a substrate for BAG6-mediated quality control. An siRNA-mediated knockdown of BAG6 strongly enhances the steady-state level of OPG–TASK_85_ (Fig. 3A, cf. lanes 4 and 5) but, as with SGTA coexpression, there is little effect upon the R4 variant that lacks a functional hydrophobic degron (Fig. 3A, cf. lanes 6 and 7). The actions of the BAG6 complex rely on its ability to promote the polyubiquitylation of its substrates (Hessa et al., 2011; Minami et al., 2010); hence, replacing the lysine residues present in OPG–TASK_85_ both enhances steady-state levels of the resulting OPG–TASK_85_ ΔK in comparison to the parental OPG−TASK_85_ and negates the effect of a BAG6 knockdown (Fig. 3A, cf. lanes 4, 5, 8 and 9). We conclude that OPG−TASK_85_ is normally degraded through a pathway that requires the recognition of a contiguous stretch of hydrophobicity, is facilitated by the BAG6 complex and involves the ubiquitylation of its lysine side chains. A direct role for the BAG6 subunit of the BAG6 complex in triaging OPG–TASK_85_ was underlined by a physical interaction between these two components that was not observed with OPG–TASK_85_ R4 (see Fig. 3B,C, cf. lanes 5 to 8). Furthermore, when the BAG6 subunit is exogenously expressed in HeLaM cells, a substantial proportion of the protein is found in the nucleus (Manchen and Hubberstey, 2001), resulting in a parallel redistribution of OPG–TASK_85_ from the cytosol to the nucleus of cells that coexpress both components (Fig. 3Di). Nuclear relocalisation of OPG–TASK_85_ is not observed with a BAG6 mutant bearing a non-functional nuclear localisation signal (Fig. 3Dii) or with the OPG–TASK_85_ R4 variant (Fig. 3Diii).

### SGTA overexpression delays the proteasomal degradation of BAG6 substrates

**Fig. 4.**
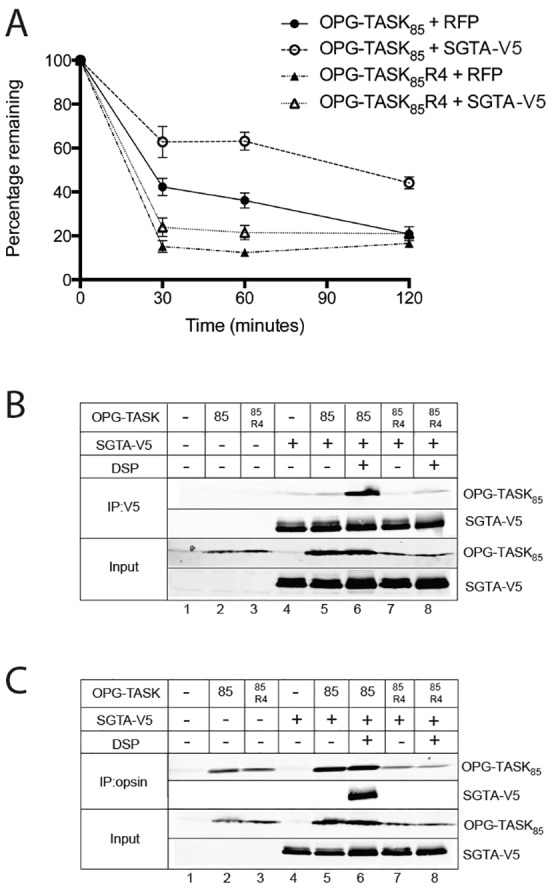
**OPG–TASK_85_ is protected from degradation by exogenous SGTA.** (A) Cells were co-transfected with plasmids encoding OPG–TASK_85_ or OPG–TASK_85_ R4 and SGTA or RFP, as indicated, and 24 hours later, 100 µg/ml cycloheximide was added to the medium and the cells were harvested directly into sample buffer at the indicated times. The amount of OPG–TASK_85_ derivative present at each time-point was determined by quantitative western blotting, and the resulting value was expressed as a percentage of the initial level obtained at 0 minutes (see also supplementary material Fig. S2A). Data show the mean±s.e.m. (*n* = 3). (B,C) Cells were co-transfected with either OPG–TASK_85_ or OPG–TASK_85_ R4 and SGTA–V5, as indicted, and then further processed for co-immunoprecipitation as described in the legend for Fig. 3B,C.

To better define the molecular basis for the increased steady-state levels of MLPs observed in the presence of exogenous SGTA, we analysed OPG–TASK_85_ levels over a 2-hour time course by using a cycloheximide block to inhibit new protein synthesis (Yewdell et al., 2011). This approach revealed that in control RFP-expressing cells, ∼50% of OPG–TASK_85_ was degraded in 30 minutes, whereas it took 120 minutes to achieve a similar reduction in the presence of exogenous SGTA (Fig. 4A; supplementary material Fig. S2A). By contrast, the degradation of OPG–TASK_85_ R4 appeared to be unaffected by SGTA overexpression (Fig. 4A; supplementary material Fig. S2A). In addition to its proposed role in MLP quality control (Leznicki and High, 2012), SGTA is also implicated in ERAD, and the removal of unassembled TCRα–YFP from the ER membrane is delayed by an SGTA knockdown (Xu et al., 2012). Like the MLP OP91, the opsin degron mutant (OpD) is derived from bovine opsin, although OpD is a mutant version of full-length opsin that is a well-defined ERAD substrate (Fleig et al., 2012; Ray-Sinha et al., 2009). We recently showed that OpD degradation is facilitated by BAG6 (Payapilly and High, 2014) and therefore asked whether SGTA also plays any role in OpD quality control. To this end, we investigated the effect of SGTA overexpression on OpD stability, and found that it also resulted in a significant delay in OpD degradation (supplementary material Fig. S2B,C). We conclude that, like BAG6 (Kawahara et al., 2013), SGTA can influence the fate of a range of hydrophobic substrates, including both misfolded and mislocalised membrane proteins.

These data provide evidence that cellular SGTA levels might directly influence MLP degradation, and we explored the possibility that this reflects a physical interaction. When labile or transient protein–protein interactions were stabilised, by treating cells with the cleavable cross-linking reagent dithiobis(succinimidylpropionate) (DSP), OPG–TASK_85_ was co-immunoprecipitated with SGTA from the resulting cell extracts, strongly suggesting a direct interaction between these two components (Fig. 4B,C; lane 6). However, no such interaction was apparent with OPG–TASK_85_ R4 (Fig. 4B,C; lane 8), underlining the importance of substrate hydrophobicity for the binding of MLPs to SGTA (supplementary material Fig. S1B). Using a complementary pull-down approach, the ability of *in*-*vitro*-synthesised OPG–TASK_85_ to bind to recombinant SGTA was also analysed (Leznicki et al., 2011; Leznicki et al., 2013). OPG–TASK_85_ showed robust and specific binding to SGTA (supplementary material Fig. S2D, cf. lanes 2 and 5 of pulldown), whereas OPG–TASK_85_ R4 did not (supplementary material Fig. S2D, cf. lanes 3 and 6). In short, the substrate specificity of SGTA shows a striking resemblance to that of BAG6, and the two components appear to cooperate to control the proteasomal degradation of MLPs.

### SGTA regulates MLP polyubiquitylation

**Fig. 5.**
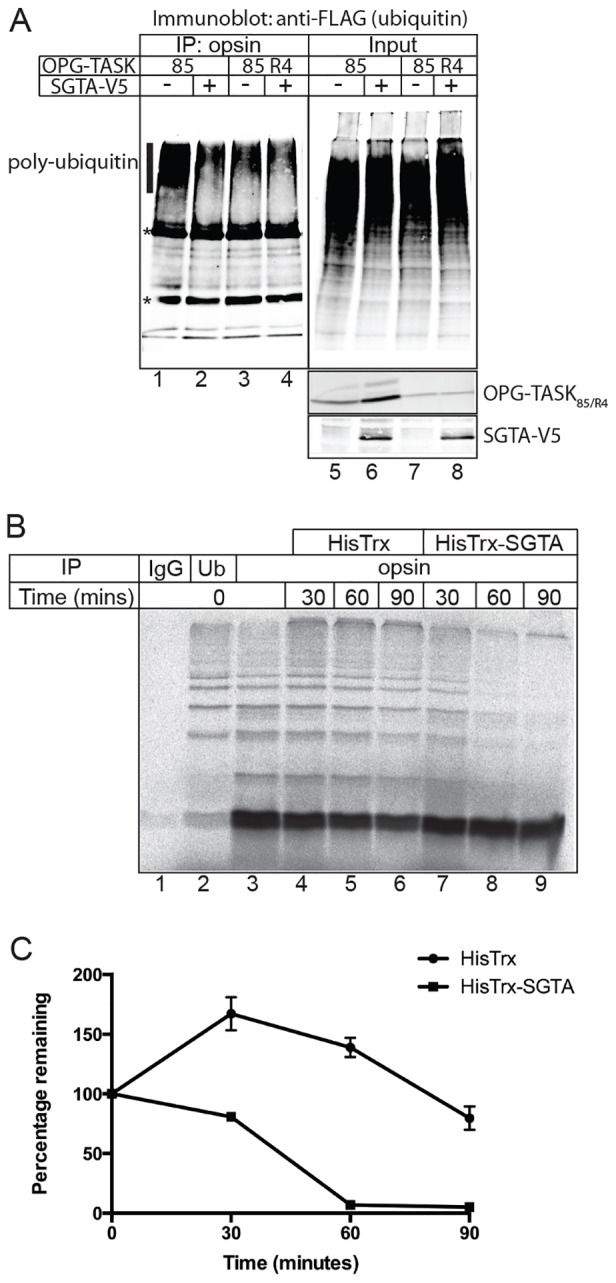
**SGTA promotes deubiquitylation of MLPs.** (A) Cells were co-transfected with plasmids encoding OPG–TASK_85_ or OPG–TASK_85_ R4, FLAG–ubiquitin and either SGTA–V5 (+) or RFP (−), as shown. After 24 hours, the cells were lysed in RIPA buffer, specific products recovered by immunoprecipitation (IP) with anti-opsin, and the resulting samples analysed for FLAG–ubiquitin by western blotting (lanes 1–4). A 10% fraction of each input was analysed in parallel (lanes 5–8). Bands corresponding to polyubiquitylated products (vertical line) and antibody chains (*) are indicated. (B) OPG–TASK_85_ was synthesised using rabbit reticulocyte lysate and, after termination of further translation initiation using ATCA, 2 µM of either recombinant HisTrx–SGTA or HisTrx control was added and samples were incubated for the times indicated before NEM was added to prevent any further modifications. Samples were then treated with DTT and pre-cleared in immunoprecipitation buffer containing 1% Triton X-100 using pansorbin. After centrifugation to clarify, immunoprecipitation was performed overnight with Protein-A–Sepharose and the indicated antibodies or an IgG control. The resin was washed and samples were analysed by SDS-PAGE and phosphorimaging. (C) High-molecular-mass species recovered following immunoprecipitation with the anti-opsin antibody were quantified for each time-point shown in B, and the resulting values were expressed as a percentage of the signal recovered at time 0. Data show the mean±s.e.m. (*n* = 3).

Our working hypothesis was that SGTA acts by regulating the polyubiquitylation status of MLPs (Leznicki and High, 2012). When OPG–TASK_85_ was coexpressed with a FLAG-tagged version of ubiquitin and the MLP substrate then recovered by immunoprecipitation, high-molecular-mass FLAG–ubiquitin-containing species were readily apparent, consistent with the efficient polyubiquitylation of OPG–TASK_85_ (Fig. 5A, lane 1). The level of these polyubiquitin–OPG–TASK_85_ conjugates showed a noticeable qualitative reduction upon SGTA coexpression (Fig. 5A, cf. lanes 1 and 2). By contrast, SGTA coexpression had a more modest qualitative effect upon the level of polyubiquitylated OPG–TASK_85_ R4 recovered under identical conditions (Fig. 5A, cf. lanes 3 and 4). The addition of recombinant SGTA to an established cell-free system for studying the polyubiquitylation of BAG6 substrates (Leznicki and High, 2012) also resulted in a marked decrease in the extent of OPG–TASK_85_ polyubiquitylation (supplementary material Fig. S2E, cf. lanes 2–4). Furthermore, when this *in vitro* system was employed to enable a ‘pulse-chase’ style analysis of substrate polyubiquitylation, the addition of exogenous SGTA was found to promote the deubiquitylation of previously modified OPG–TASK_85_ chains (Fig. 5B, cf. lanes 4–6 and 7–9; Fig. 5C). Taken together, these data support the hypothesis that SGTA overexpression results in a substantial increase in the steady-state level of MLPs by promoting their deubiquitylation and, hence, inhibiting their proteasomal degradation.

### SGTA-dependent stabilisation results in substrate aggregation

**Fig. 6.**
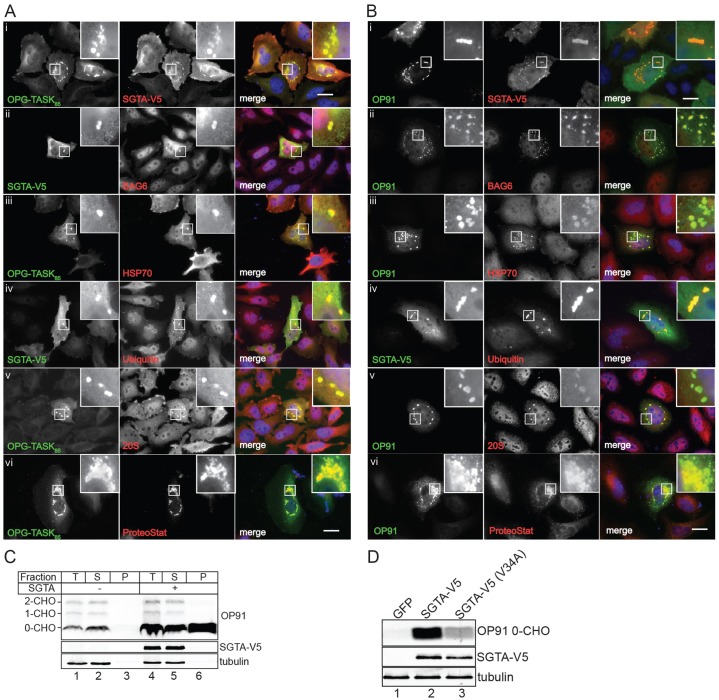
**SGTA promotes the aggregation of mislocalised proteins.** (Ai–v) HeLa Flp-In T-REx cells that stably express OPG–TASK_85_ under the control of a tetracycline-inducible promoter were transiently transfected with SGTA–V5 and induced to express OPG–TASK_85_. (Bi–v) HeLaM cells were co-transfected with plasmids encoding OP91 and SGTA–V5. For A and B, at 24 hours post-transfection, samples were processed for microscopy to examine the subcellular localisation of the relevant MLP (anti-opsin), exogenous SGTA (anti-V5) and various endogenous components, including BAG6 (ii), Hsp70 (iii), ubiquitin (iv) and the 20S proteasomal subunit (v), and visualised by immunofluorescence. (Avi,Bvi) HeLaM cells were co-transfected with plasmids encoding OPG–TASK_85_ or OP91 and SGTA–V5, and stained using an anti-opsin antibody to detect MLP substrates and ProteoStat® to visualise protein aggregates. Enlarged sections (insets) highlight cytosolic inclusions that resemble aggresomes (see text). Scale bars: 20 µm. (C) HeLaM cells were co-transfected with plasmids encoding OP91 and RFP (lanes 1–3) or SGTA–V5 (lanes 4–6) and, 24 hours later, they were solubilised in buffer containing 1% Triton X-100. The resulting lysate was centrifuged to separate soluble and insoluble material and then equivalent amounts of supernatant (S) and pellet (P), together with 10% of the total lysate (T), were analysed by western blotting as described for Fig. 2. (D) HeLaM cells were co-transfected with plasmids encoding OP91 and GFP (lane 1), SGTA–V5 (lane 2) or SGTA–V5(V34A) (lane 3) and, after 24 hours, were further processed for western blotting as before.

When cells expressing either OPG–TASK_85_ or OP91 were studied using immunofluorescence microscopy, discrete intracellular punctae were observed in the presence of exogenous SGTA (Fig. 6Ai,Bi). These putative cytosolic inclusions contained both the relevant MLP substrate, be it OPG–TASK_85_ or OP91, and SGTA (Fig. 6Ai,Bi, see merge), and were not apparent when the MLPs or SGTA were expressed alone (supplementary material Fig. S3A,B), suggesting that their formation might be substrate driven. SGTA overexpression also resulted in the appearance of large inclusions that contained both exogenous SGTA and the ERAD substrate OpD (supplementary material Fig. S3C,D), further suggesting that SGTA influences the cellular fate of both MLPs and ERAD substrates. In the case of the MLPs, these SGTA-dependent punctae were further characterised and found to be positive for endogenous BAG6 and HSP70 (Fig. 6Aii,iii,Bii,iii), consistent with a role in quality control, and they also contained both ubiquitin and proteasomes (Fig. 6Aiv,v,Biv,v), supporting the suggestion that these structures are inclusion bodies or aggresomes (Hao et al., 2013). To further explore the nature of these structures, we made use of ProteoStat®, a fluorescent red dye that is only detectable when it is bound to aggregated proteins (Shen et al., 2011). Many of the large punctate structures observed upon expression of MLPs in the presence of exogenous SGTA were strongly labelled with ProteoStat®, further suggesting that they contain protein aggregates. By contrast, the cytosolic ER delivery factor TRC40 was not detected in the MLP-positive cytosolic inclusions (supplementary material Fig. S4A), and there was little overlap between SGTA-positive punctae and the lysosomal marker LAMP1 (supplementary material Fig. S4B), consistent with the inability of lysosomal protease inhibitors to prevent MLP degradation (supplementary material Fig. S1C). The OP91-positive inclusions observed upon SGTA overexpression were also clearly distinct from both the ER and Golgi, consistent with a cytosolic localisation (supplementary material Fig. S4C).

Aberrant and misfolded precursor proteins are often aggregation prone (Ast and Schuldiner, 2011; Ast et al., 2013), and we investigated the status of SGTA-stabilised MLPs using selective detergent solubilisation. Under control conditions, all detectable OP91 was recovered in a Triton-X-100-soluble fraction (Fig. 6C, cf. lanes 2 and 3), yet we found that a substantial proportion of the non-glycosylated form of OP91 was found in the insoluble pellet fraction upon SGTA coexpression (Fig. 6C, cf. lanes 5 and 6, see OP91 0-CHO). Such behaviour is strongly indicative of cytosolic aggregation (Chakrabarti et al., 2011) and, together with our imaging data, suggests that the cytosolic inclusions that we observe in the presence of exogenous SGTA represent aggresomes or aggresome-like structures (Rodriguez-Gonzalez et al., 2008).

Recent structural studies have revealed a central role for the N-terminal region of SGTA in facilitating its binding to the BAG6 complex, through its UBL-domain-containing subunits (Chartron et al., 2012; Simon et al., 2013; Xu et al., 2012). Because our data show that both BAG6 and SGTA bind to MLPs and colocalise to cytoplasmic MLP-containing inclusions upon SGTA overexpression, we asked whether the BAG6 complex contributes to the SGTA-dependent stabilisation of MLPs. Several studies have defined the molecular basis for the binding of specific UBLs to SGTA family members (Chartron et al., 2012; Simon et al., 2013; Xu et al., 2012), and we chose to make a V34A variant of SGTA that preserves its dimerisation interface whilst reducing its affinity for cognate UBLs by approximately two orders of magnitude (see Chartron et al., 2012; Simon et al., 2013). Strikingly, although the V5-tagged SGTA(V34A) variant was expressed at a level comparable to that of the parental SGTA–V5, its ability to enhance steady-state MLP levels was greatly reduced (Fig. 6D). These data strongly suggest that the stabilisation of MLPs observed upon SGTA overexpression is dependent upon efficient binding of exogenous SGTA to the endogenous BAG6 complex. On the basis of these data, we conclude that MLP substrates, exemplified here by OPG–TASK_85_ and OP91, enter a cytosolic quality control pathway that is mediated by the BAG6 complex and SGTA working in tandem (Hessa et al., 2011; Kawahara et al., 2013; Leznicki et al., 2013).

## DISCUSSION

**Fig. 7.**
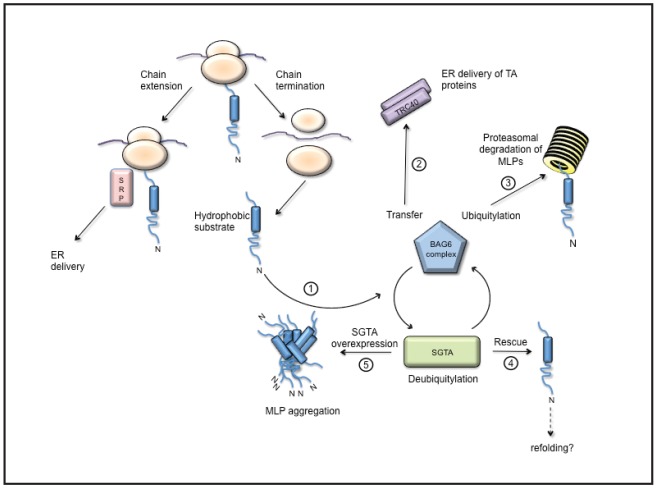
**A potential BAG6/SGTA cycle for cytosolic quality control.** Hydrophobic polypeptides that fail to engage SRP, including tail-anchored (TA) proteins and short fragments of integral membrane proteins, become substrates for both SGTA and the BAG6 complex (step 1). Tail-anchored proteins are most likely passed from SGTA to the BAG6 complex before specific transfer to the ER delivery factor TRC40 (step 2; see Chartron et al., 2012; Leznicki et al., 2010; Mariappan et al., 2010). By contrast, the binding of MLPs to the BAG6 complex promotes their ubiquitylation (Rodrigo-Brenni et al., 2014) and proteasomal degradation (step 3), a process that is antagonised by SGTA (Leznicki and High, 2012). We postulate that SGTA normally acts to provide a rescue pathway for prematurely ubiquitylated substrates (step 4), thereby prolonging their opportunity for successful ER delivery and/or native folding. SGTA overexpression causes the inhibition and/or reversal of BAG6-mediated MLP ubiquitylation (step 5), resulting in their accumulation and aggregation.

When Sec61-mediated protein translocation at the ER is inhibited, for example using a small molecule inhibitor or following a stress response, the resulting non-translocated membrane and secretory proteins mislocalise to the cytosol and are rapidly degraded unless proteasomal degradation is blocked (Besemer et al., 2005; Kang et al., 2006). Furthermore, there is good evidence that regions of exposed hydrophobicity, including unembedded transmembrane domains act as a potent signal to elicit the degradation of such MLPs (Huang et al., 2011). Recent studies suggest that MLP degradation is selective, and they have directly implicated two key factors, SGTA (Leznicki and High, 2012) and the BAG6 complex (Hessa et al., 2011), as mediators of this process (Fig. 7). Here, we have developed model MLPs and exploited them to study the role of SGTA during MLP quality control.

### SGTA binds to MLPs

Our studies show that SGTA binds directly to MLPs, both *in vitro* and *in vivo*, and underline the importance of an uninterrupted hydrophobic degron for this interaction to occur. Crucially, the ability of an aberrant protein to bind to SGTA directly impacts on the role that SGTA plays in its quality control, strongly suggesting that its actions are direct. Most strikingly, although OPG–TASK_85_ R4 is clearly a substrate for proteasomal degradation, and hence is stabilised by the proteasome inhibitor bortezomib, it does not associate with SGTA and its stability is unaffected by either increasing or decreasing the level of SGTA. By contrast, OPG–TASK_85_ shows a robust interaction with SGTA and its steady-state expression level and stability are directly correlated with the amount of SGTA present in a cell. Reductions in SGTA by siRNA treatment reduce OPG–TASK_85_ levels, whereas exogenous expression of SGTA has the opposite effect. These findings extend the substrate base for SGTA from the *in vitro* binding of tail-anchored membrane proteins and the glucose transporter (Leznicki et al., 2010; Leznicki et al., 2011; Liou and Wang, 2005) to include the *in vivo* binding of a model MLP.

### SGTA substrate specificity overlaps with that of the BAG6 complex

There is extensive evidence for a physical interaction between SGTA and the BAG6 complex (see Chartron et al., 2012; Leznicki et al., 2013; Simon et al., 2013; Xu et al., 2012 and references therein), as well as evidence that exposed hydrophobicity is an important feature of protein substrates that are dealt with by the BAG6 complex (Kawahara et al., 2013). Strikingly, the *in vivo* interactions of MLPs with the BAG6 protein mirror those of SGTA, hence BAG6 binds to OPG–TASK_85_ but not to OPG–TASK_85_ R4. This substrate specificity is very clearly demonstrated by the ability of exogenous BAG6 to relocalise OPG–TASK_85_ to the nucleus, a finding that mirrors the BAG6-dependent nuclear relocalisation of the ERAD substrate OpD (Payapilly and High, 2014). Taken together, these data are consistent with a model where hydrophobic substrates located in the cytosol can be passed between SGTA and the BAG6 complex (Fig. 7), a process that might be facilitated by a fast exchange between these two cellular quality control components (Chartron et al., 2012). One important, but as yet unanswered, question is how SGTA and BAG6 are able to distinguish different classes of hydrophobic substrate and deal with them appropriately. Hence, whereas tail-anchored membrane proteins are passed to TRC40 for subsequent ER delivery, MLPs and ERAD substrates are normally efficiently degraded at the proteasome (Fig. 7). The molecular basis for this apparent substrate selectivity is currently unclear.

### SGTA regulates MLP degradation

We now show that the cellular basis for the previously reported increase in steady-state MLPs following SGTA overexpression (Leznicki and High, 2012) is a consequence of the selective inhibition of their proteasomal degradation. Furthermore, we find that SGTA overexpression also delays the proteasomal degradation of the ERAD substrate OpD, confirming that, like BAG6 (Kawahara et al., 2013), SGTA contributes to the quality control of a range of hydrophobic substrates. Although we find that SGTA overexpression stabilises both a model MLP and an ERAD substrate, it remains to be established whether the physiological role of SGTA during these two processes is comparable (Fig. 7). Interestingly, several recent reports correlate increases in SGTA expression with the proliferation of several forms of cancer, although the molecular basis for this observation remains to be defined (Lu et al., 2014; Philp et al., 2013; Xue et al., 2013).

On the basis of the data presented in this study, we propose that the molecular mechanism underlying the increase in steady-state MLP levels observed upon SGTA overexpression occurs through an effect on MLP polyubiquitylation status, with increased levels of SGTA favouring the deubiquitylated form of MLPs both *in vitro* and *in vivo*. Taken in a wider context, these data support the operation of a cytosolic quality control cycle for hydrophobic substrates (Fig. 7). This cycle would be driven by the opposing actions of SGTA and the BAG6 complex, a proposal that is supported by our finding that for exogenous SGTA to stabilise MLPs it must be able to interact with the endogenous BAG6 complex (Fig. 6D). We favour a model where an artificial increase in the level of SGTA promotes substrate deubiquitylation, and our *in vitro* data support this proposal (Fig. 5B; Leznicki and High, 2012). However, the binding of exogenous SGTA to MLP substrates and/or the BAG6 complex might also act to directly inhibit their ubiquitylation *in vivo* (Fig. 7) through a pathway that almost certainly involves the E3 ligase RNF126 (Rodrigo-Brenni et al., 2014). Our current hypothesis is that the pronounced stabilisation of MLPs observed upon SGTA overexpression provides a non-physiological example of a ‘rescue’ pathway that would normally favour certain hydrophobic substrates such as tail-anchored membrane proteins. In this scenario, the possibility to deubiquitylate precursor proteins that undergo premature ubiquitylation could prolong their window of opportunity for post-translational membrane integration (Fig. 7; cf. Leznicki and High, 2012). Interestingly, a comparable cycle of ubiquitylation and deubiquitylation has been suggested to provide quality control for hydrophobic GPI-anchored protein precursors that fail to engage the ER translocation machinery in *Saccharomyces cerevisiae* (Ast et al., 2014). Deubiquitylases can also enhance the substrate selectivity of proteasomal degradation (Liu et al., 2014; Zhang et al., 2013) and, in this context, a BAG6/SGTA-dependent cycle of ubiquitylation and deubiquitylation might fine-tune the fate of distinct hydrophobic substrates that become exposed to the cytosol (Fig. 7).

### SGTA overexpression promotes protein aggregation

Having established that SGTA overexpression inhibits the proteasomal degradation of aberrant membrane proteins, we explored the intracellular fate of these aberrant polypeptides. Immunofluorescence microscopy revealed that both our model MLPs and ERAD substrate are localised to discrete intracellular punctae when coexpressed with SGTA but not in its absence. In the case of the ERAD substrate OpD, these structures are comparable to those observed with TCRα–YFP upon SGTA depletion (Xu et al., 2012). In the case of the MLPs, these cytoplasmic punctae co-stain for both exogenous SGTA and endogenous BAG6; in addition to Hsp70 chaperones, ubiquitin and 20S proteasomal subunits, suggesting that they represent aggregated MLPs or aggresomes. This proposal is further supported by our observations that SGTA-stabilised forms of OPG–TASK_85_ and OP91 are selectively stained with ProteoStat®, whilst a substantial proportion of SGTA-stabilised OP91 is also recovered in a detergent-insoluble form, both strongly indicative of protein aggregation (Chakrabarti et al., 2011; Shen et al., 2011). On the basis of these data, we tentatively suggest that the MLP-containing punctae observed upon SGTA overexpression represent structures that are related to cytoplasmic juxtanuclear quality control compartments (Ben-Gedalya and Cohen, 2012). These are dynamic sites for cellular quality control where substrates might be either deubiquitylated and subjected to chaperone-mediated refolding, or ubiquitylated and directed to the proteasome for degradation (Ben-Gedalya and Cohen, 2012). Our data suggest that an unperturbed BAG6/SGTA quality control cycle promotes the biogenesis of selected hydrophobic precursors at the ER, whilst also facilitating the removal of aberrant and misfolded membrane proteins. Together, these actions will decrease the opportunities for such hydrophobic proteins to form cytosolic aggregates and reduce their potential impact upon cellular proteostasis (Buchberger et al., 2010; Chakrabarti and Hegde, 2009; Hartl et al., 2011; Park et al., 2013; Fig. 7).

## MATERIALS AND METHODS

### Materials

All cell culture and standard reagents were purchased from Sigma. The region of bovine opsin used as an N-glycosylation reporter and epitope tag was as described previously (Johnson et al., 2012). Anti-TRC40 serum was a gift from Bernhard Dobberstein (ZMBH, Heidelberg, Germany). Commercially available antibodies against the following targets were purchased from the indicated suppliers: BAG6, BAP31, GFP, tubulin and V5 (Abcam), FLAG M2 and calnexin (Sigma), HSP70 (Stressgen), ubiquitin FK2, p23, 20S proteasome (Enzo Life Sciences) and LAMP1 (DHSB, University of Iowa). The antibody against GRASP65 was a gift from Martin Lowe (University of Manchester, UK). ProteoStat® reagent for the detection of protein aggregates was from Enzo. Bortezomib was from Selleck Chemicals, leupeptin and pepstatin A were from BIOMOL. RFP in pcDNA3.1^+^ was a gift from Viki Allan (Manchester, UK). siRNA duplexes for knockdowns were from Qiagen, and they targeted sequences that were identified previously (Winnefeld et al., 2006): SGTA target sequence, 5′-TTTGAAGCTGCCGTGCATT-3′; BAG6 target sequence, 5′-CAGCTCCGGTCTGATATACAA-3′.

### Plasmids

Fragments of TASK-1 without cysteine residues (C14V), TASK_85_ and TASK_100_ were subcloned, with the first 26 amino acids of bovine opsin at the N-terminus, into pcDNA3.1^+^ (Life Technologies), generating OPG–TASK constructs. OPG–TASK_85_ was also subcloned into pcDNA5/FRT/TO (Life Technologies). SGTA and BAG6 were in pcDNA5/FRT/V5-His-TOPO in frame to add the V5 tag. OP91, OP91CHO and eGFP were in the same vector, but with stop codons prior to the tag. Ub-R–GFP was obtained through Addgene (Dantuma et al., 2000). The FLAG–ubiquitin construct was a gift from Sylvie Urbé (University of Liverpool, UK). BAG6 ΔNLS, SGTA V34A, OPG–TASK_85_ R4 and the lysine-deficient mutant were generated by using the QuikChange site-directed mutagenesis kit (Stratagene).

### Cell culture

HeLa and HeLaM cells were maintained in DMEM containing 10% fetal bovine serum and 2 mM L-glutamine at 37°C under 5% CO_2_. DNA transfections were performed using Lipofectamine 2000 (Life Technologies) for 24 hours in accordance with the manufacturer's instructions. For siRNA experiments, 20 nM siRNA duplexes were transfected using INTERFERin (Polyplus) as specified by the manufacturer. The cells were then transiently transfected with the appropriate DNA constructs after 48 hours and harvested for analysis after 72 hours. The inducible stable HeLa cell line expressing OpD was as described previously (Payapilly and High, 2014), whereas the OPG–TASK_85_ was generated for this study using HeLa Flp-In T-REx cells, which were a gift from Stephen Taylor (University of Manchester, UK; Tighe et al., 2008). For drug treatments, 10 nM bortezomib, 100 µM leupeptin and 1 µg/ml pepstatin were added at 18 hours prior to analysis.

### Subcellular fractionation

Transfected HeLaM cells were lysed mechanically using a ball-bearing cell cracker with 10-µm clearance in buffer containing 20 mM HEPES-KOH pH 7.4, 100 mM KCl and 5 mM MgCl_2_. Unbroken cells and large debris were removed by centrifugation at 2000 ***g*** for 10 minutes. The membrane and cytosolic fractions were separated by ultra-centrifugation at 100,000 ***g*** for 10 minutes at 4°C.

### Western blotting

Samples were prepared for western blotting and specific proteins were detected as described previously (Leznicki and High, 2012). Quantification was performed using Image Studio (Li-Cor Biosciences) employing data from at least three independent experiments. When used, endoglycosidase H (New England Biolabs) was added at 20 units/µl directly to material in SDS sample buffer and samples were incubated at 37°C for at least 3 hours.

### Co-immunoprecipitation

Co-transfected HeLaM cells were solubilised in 10 mM Tris-HCl pH 7.4, 150 mM NaCl and 1 mM EDTA supplemented with protease inhibitor cocktail, 1 mM PMSF and 0.5% n-dodecyl β-D-maltopyranoside (DDM) for 1 hour at 4°C. Where indicated, cells were treated with the cleavable cross-linking reagent dithiobis(succinimidylpropionate) (DSP, Pierce) at 1 mM for 15 minutes at room temperature and then quenched with 10 mM glycine prior to the addition of the DDM lysis buffer. To recover FLAG-tagged ubiquitin conjugates, cells were lysed in RIPA buffer (50 mM Tris-HCl pH 7.4, 150 mM NaCl, 2 mM EDTA, 1% NP-40, 0.5% sodium deoxycholate and 0.1% SDS) containing protease inhibitors, 1 mM PMSF and 20 mM N-ethylmaleimide (NEM) and then sonicated. Insoluble material was removed by centrifugation at 16,000 ***g*** for 10 minutes at 4°C, 10% of the supernatant was retained as the input sample, and the remainder used for the immunoprecipitation of specific proteins using an appropriate antibody and Protein-A–Sepharose (Genscript).

### Cycloheximide chase and *in vitro* ubiquitylation assay

The stability of OPG–TASK derivatives was studied by treating transfected HeLaM cells with 100 µg/ml cycloheximide (Sigma) and lysing them directly in SDS-PAGE sample buffer at the indicated time-points prior to analysis by western blotting. For OpD, a stable inducible cell line was transfected with plasmids encoding GFP or V5-tagged SGTA, and samples were processed as described previously (Payapilly and High, 2014). *In vitro* ubiquitylation of OPG–TASK derivatives in the presence of HisTrx or HisTrx–SGTA was performed as described previously (Leznicki and High, 2012). AIDA software was used to quantify the resulting polyubiquitylated material.

### *In vitro* SGTA-binding assay

OPG–TASK_85_ or OPG–TASK_85_ R4 were translated in reticulocyte lysate reactions containing ^35^S-methionine/cysteine and supplemented with 2 µM HisTrx–SGTA or HisTrx (Leznicki and High, 2012). To terminate the translation, samples were incubated in the presence of 1 mM puromycin for 10 minutes at 30°C. A 10% sample of the input was retained for analysis, and the remaining reaction was diluted to give a final volume of 150 µl with buffer containing 50 mM HEPES-KOH pH 7.5, 300 mM NaCl, 50 mM imidazole, 10% glycerol and 10 µl (bead volume) Ni-NTA agarose (Qiagen). After incubation for 2 hours at 4°C with shaking, the agarose was washed three times with 1 ml of buffer as above, before elution in 30 µl of buffer containing 500 mM imidazole. The resulting samples were analysed by SDS-PAGE, and radiolabelled products were detected by phosphorimaging.

### Selective detergent solubility analysis

Transfected HeLaM cells were solubilised in 10 mM Tris-HCl pH 7.4, 150 mM NaCl, 1 mM EDTA containing 1% (v/v) Triton X-100, protease inhibitor cocktail and 1 mM PMSF for 30 minutes at 4°C. A 10% input sample was taken and the soluble and insoluble materials were isolated by centrifugation at 16,000 ***g*** for 30 minutes at 4°C.

### Immunofluorescence microscopy

Cells grown on glass coverslips were transiently transfected using jetPEI (Polyplus) for HeLaM cells (OP91 expression) and Fugene HD (Promega) for the OPG–TASK_85_ and OpD stable cell lines, which were also induced for 16–24 hours with 1 µg/ml tetracycline prior to analysis. For ProteoStat® staining, HeLaM cells were co-transfected with the SGTA–V5 construct and plasmids encoding OPG–TASK_85_ or OP91 using Fugene HD. Fixation was performed using 4% paraformaldehyde, and cells were permeabilised with 0.1% Triton X-100, except for LAMP1 staining where methanol fixation (−20°C, 5 minutes) was performed. Alexa-Fluor-488-conjugated and Alexa-Fluor-594-conjugated secondary antibodies were from Jackson ImmunoResearch, the ProteoStat® reagent for detection of protein aggregates was from Enzo Life Sciences and DNA was stained using 4′,6-diamidino-2-phenylindole (DAPI). Coverslips were mounted using Prolong Gold (Life Technologies) and fluorescence was visualised using a wide-field Olympus BX-60 microscope with a 60×/1.40 NA PlanApo objective and a CoolSnap ES camera (Roper Scientific), with images captured using MetaVue software.

## Supplementary Material

Supplementary Material
