# Supplementary material for: SGTA regulates the cytosolic quality control of hydrophobic substrates
Source: J Cell Sci. 2014 Nov 1;127(21):4728–39. doi: 10.1242/jcs.155648 (PMC4215715; doi:10.1242/jcs.155648)
Supplement: Supplementary Material [file supp_127_21_4728__index.html]

SGTA regulates the cytosolic quality control of hydrophobic substrates — Supplementary Material 

# SGTA regulates the cytosolic quality control of hydrophobic substrates

## JCS155648 Supplementary Material

**Files in this Data Supplement:**

- **Supplementary Material**
