# Supplementary material for: SGTA regulates the cytosolic quality control of hydrophobic substrates
Source: J Cell Sci. 2014 Nov 1;127(21):4728–39. doi: 10.1242/jcs.155648 (PMC4215715; doi:10.1242/jcs.155648)
Supplement: Supplementary Material [file supp_127.21.4728_JCS155648.pdf]

SUPPLEMENTARY FIGURES

SGTA regulates the cytosolic quality control of hydrophobic substrates

Wunderley, L., Leznicki, P., Payapilly, A. and High. S.

Figure S1. The effect of SGTA on steady state MLP levels.

A) Schematic of OPG-TASK$_{85}$. Residues 1-26 of bovine opsin constitute the N-terminal OPG tag employed in this study, whilst the remaining sequence is from a cys-null version of the human TASK-1. The sole hydrophobic transmembrane region present in this chimera is shown in white, and the residues in bold indicate those substituted for arginine in the R4 variant. The OPG-TASK$_{85}$ ΔK variant was made by substituting lysines 16 and 27 of OPG-TASK$_{85}$ for arginine. **B**) Hydrophobicity plots of OPG-TASK$_{85}$ and OPG-TASK$_{85}$ R4 generated using http://dgpred.cbr.su.se/. **C**) HeLa cells transfected with OP91 were treated with either 10 nM bortezomib or 100 μM leupeptin and 1 μg/ml pepstatin or DMSO as a control for 18 hours prior to the preparation of total cell extracts and analysis by western blotting. **D**) Lysates from cells co-transfected with OP91 and either SGTA-V5 or empty vector were prepared after 24 hours and treated with EndoH or a buffer control as shown prior to analysis as for C). Levels of OP91 were quantified from 3 independent experiments and plotted showing the SEM. **E**) Cells were co-transfected with ubiquitin-R-GFP and SGTA-V5 (+) or empty vector (-) as shown and where indicated also grown in the presence of 10 nM bortezomib (+) prior to analysis as for C). HeLaM cells were transfected with a control siRNA (-) or one targeting SGTA (+). After 48 hours the cells were transfected with either **F**) OPG-TASK$_{85}$, **G**) OP91 or **H**) Ub-R-GFP, and after 72 hours the levels of each substrate were determined by western blotting.

Figure S2. SGTA delays degradation, binds MLPs and promotes deubiquitination.

A) Cells were co-transfected with plasmids encoding OPG-TASK$_{85}$ or OPG-TASK$_{85}$ R4 and SGTA-V5 or RFP as indicated and after 24 hours cycloheximide (100 μg/ml) was added to the media and cells harvested directly into sample buffer at the indicated times. The amount of OPG-TASK$_{85}$ derivative present at each time point was determined by quantitative western blotting and the resulting data used to generate Fig. 4A (see main text). **B**) HeLa Flp-In T-Rex cells that stably express OpD under the control of a tetracycline inducible promoter were either transfected with GFP (lanes 1 to 6) or SGTA-V5 (lanes 7 to 12) for 24 hours, induced to express OpD for an additional 16-20 hours, after which 100 g/ml cycloheximide was added to the media and cells harvested at the times indicated as described for A). Samples were then probed for OpD (both N-glycosylated OpD 2-CH0 and non-glycosylated OpD 0-CHO species are indicated; cf. Payapilly and High, 2014), SGTA-V5 and GFP as indicated. The asterisk indicates a cross-reacting species detected with the anti-GFP antibody. **C**) The amount of OpD 2-CHO present at each time point shown in B) was determined by quantitative western blotting and the resulting value expressed as a percentage of the initial level obtained at time 0. Error bars show SEM, n=3. **D**) OPG-TASK$_{85}$ or OPG-TASK$_{85}$ R4 were translated in rabbit reticulocyte lysate in the presence of recombinant HisTrx-SGTA of HisTrx control. A 10% sample of the input was retained for comparison and the recombinant proteins re-isolated using Ni-NTA resin. Bound proteins were eluted using imidazole and analyzed by SDS-PAGE and phosphorimaging. **E**) OPG-TASK$_{85}$ was synthesized in vitro in the presence of recombinant HisTrx-SGTA or HisTrx, specific products recovered by immunoprecipitation using antibodies specific for ubiquitin, the OPG tag or an IgG control as indicated, and samples analysed by SDS-PAGE and phosphorimaging.

Figure S3. SGTA overexpression promotes the formation of intracellular punctae

A) OPG-TASK$_{85}$ HeLa Flp-In T-REx cells were either induced with tetracycline and transfected with RFP (i), or left un-induced and transfected with SGTA-V5 (ii). **B**) HeLaM cells were co-transfected with OP91 and empty vector (i) or SGTA-V5 and GFP (ii). In all cases, cells were fixed 24 hours post-transfection and processed for immunofluorescence microscopy as indicated. OpD HeLa Flp-In T-Rex cells were transfected with empty vector (**C)** or SGTA-V5 (**D)** for 24 hours and then induced with tetracycline to express OpD for an additional 16-20 hours. Cells were fixed and labelled for OpD and the ER marker BAP31 (panel C) or OpD and SGTA-V5 (panel D). Scale bars: panels A & B = 20 µm; panels C & D = 10 µm.

Figure S4. Characterization of MLP containing cytosolic inclusions.

OPG-TASK$_{85}$ HeLa Flp-In T-REx cells were induced and transfected with SGTA-V5 and then processed for immunofluorescence microscopy and labelled for OPG-TASK$_{85}$ and the ER delivery factor, TRC40 (**A**) or SGTA-V5 and the lysosomal marker, LAMP1 (**B**). **C**) HeLaM cells were transiently transfected with plasmids encoding OP91 and SGTA-V5, processed as for A) and then labelled for OP91 and the ER marker calnexin (i) or SGTA-V5 and the Golgi marker GRASP65 (ii). Scale bar = 20 µm.

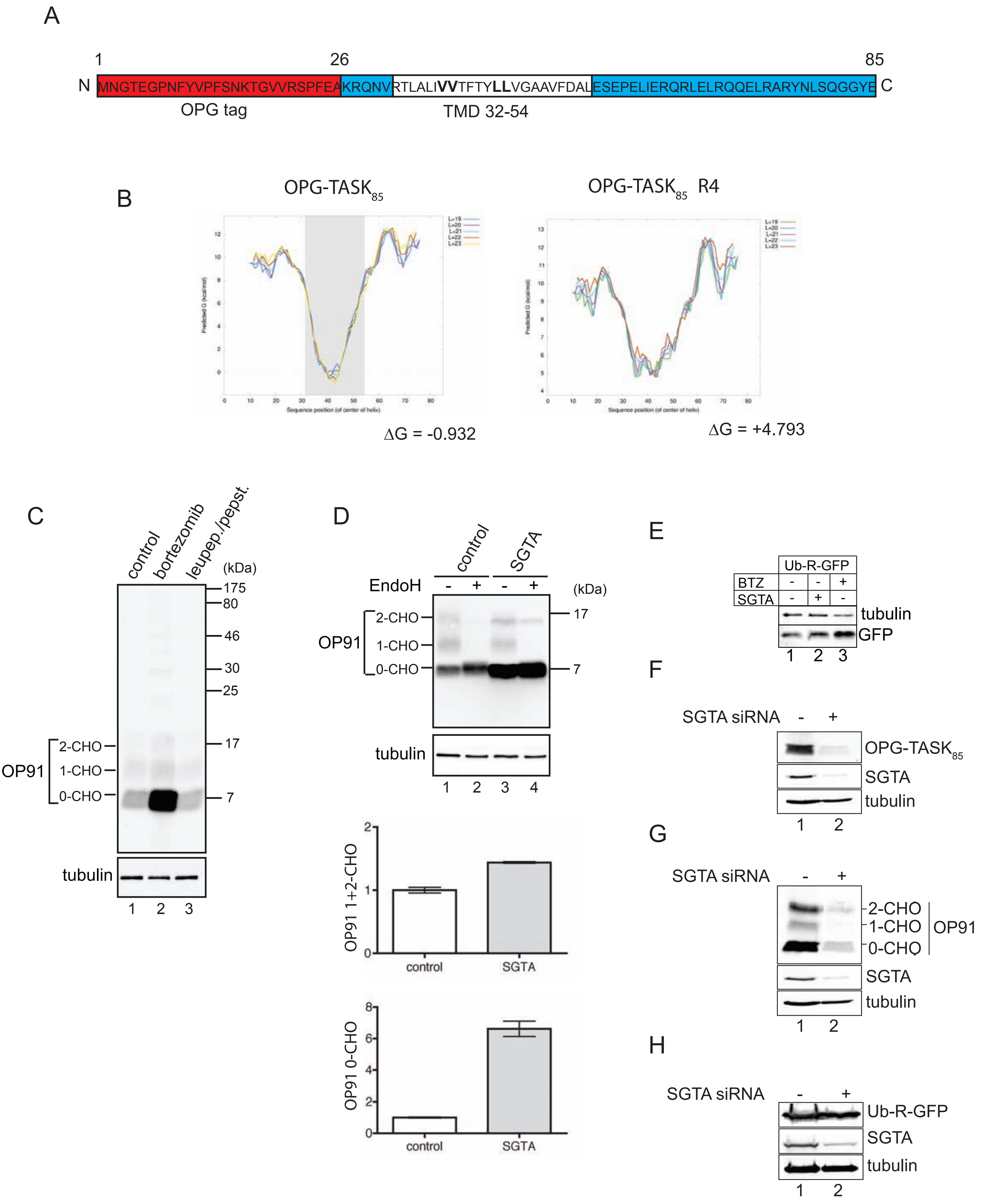
A
1
26
85
N
MNGTEGPNFYVPFSNKTGVVRSPFEAKRQNVRTLALIVVTFTYLLVGAAVFDALESEPELIERQRLELRQQELRARYNLSQGGYE
C
OPG tag
TMD 32-54
B
OPG-TASK85
OPG-TASK85 R4
Sequence position (of center of helix)
Predicted G (kcal/mol)
ΔG = -0.932
ΔG = +4.793
C
control
bortezomib
leupep./pepst.
(kDa)
175
80
46
30
25
17
7
OP91
2-CHO
1-CHO
0-CHO
tubulin
1 2 3
D
control
SGTA
EndoH
- + - +
(kDa)
17
7
OP91
2-CHO
1-CHO
0-CHO
tubulin
1 2 3 4
OP91 1+2-CHO
control
SGTA
OP91 0-CHO
control
SGTA
E
Ub-R-GFP
BTZ
SGTA
- - +
- + -
tubulin
GFP
1 2 3
F
SGTA siRNA
- +
OPG-TASK85
SGTA
tubulin
1 2
G
SGTA siRNA
- +
2-CHO
1-CHO
0-CHO
OP91
SGTA
tubulin
1 2
H
SGTA siRNA
- +
Ub-R-GFP
SGTA
tubulin
1 2

Figure S1

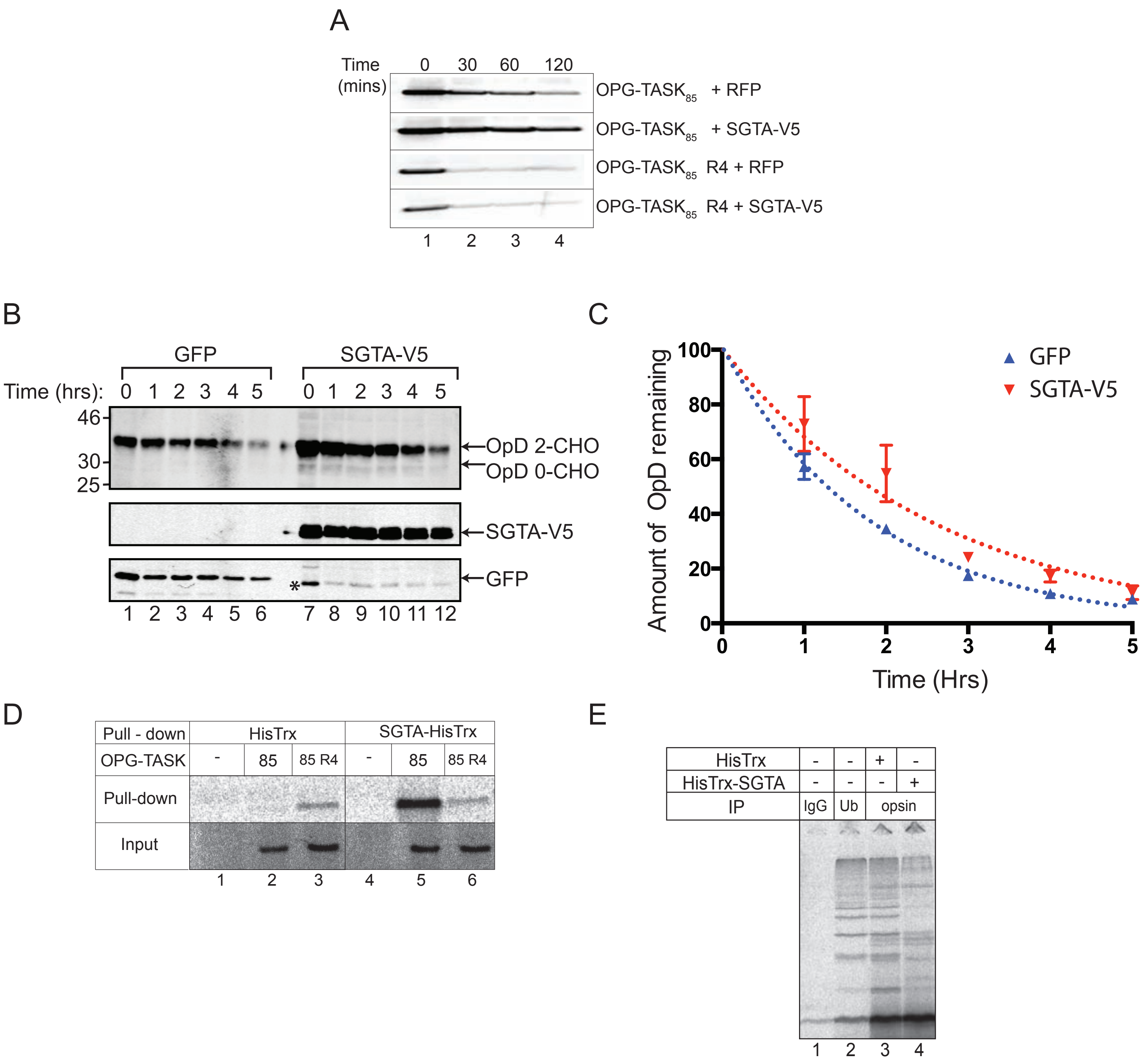
A
Time (mins)
0 30 60 120
OPG-TASK85 + RFP
OPG-TASK85 + SGTA-V5
OPG-TASK85 R4 + RFP
OPG-TASK85 R4 + SGTA-V5
1 2 3 4
B
GFP
SGTA-V5
Time (hrs): 0 1 2 3 4 5 0 1 2 3 4 5
46
30
25
OpD 2-CHO
OpD 0-CHO
SGTA-V5
GFP
*
1 2 3 4 5 6 7 8 9 10 11 12
C
GFP
SGTA-V5
Amount of OpD remaining
Time (Hrs)
D
Pull - down
HisTrx
SGTA-HisTrx
OPG-TASK
- 85 85 R4 - 85 85 R4
Pull-down
Input
1 2 3 4 5 6
E
HisTrx
HisTrx-SGTA
IP
IgG Ub opsin
1 2 3 4

Figure S2

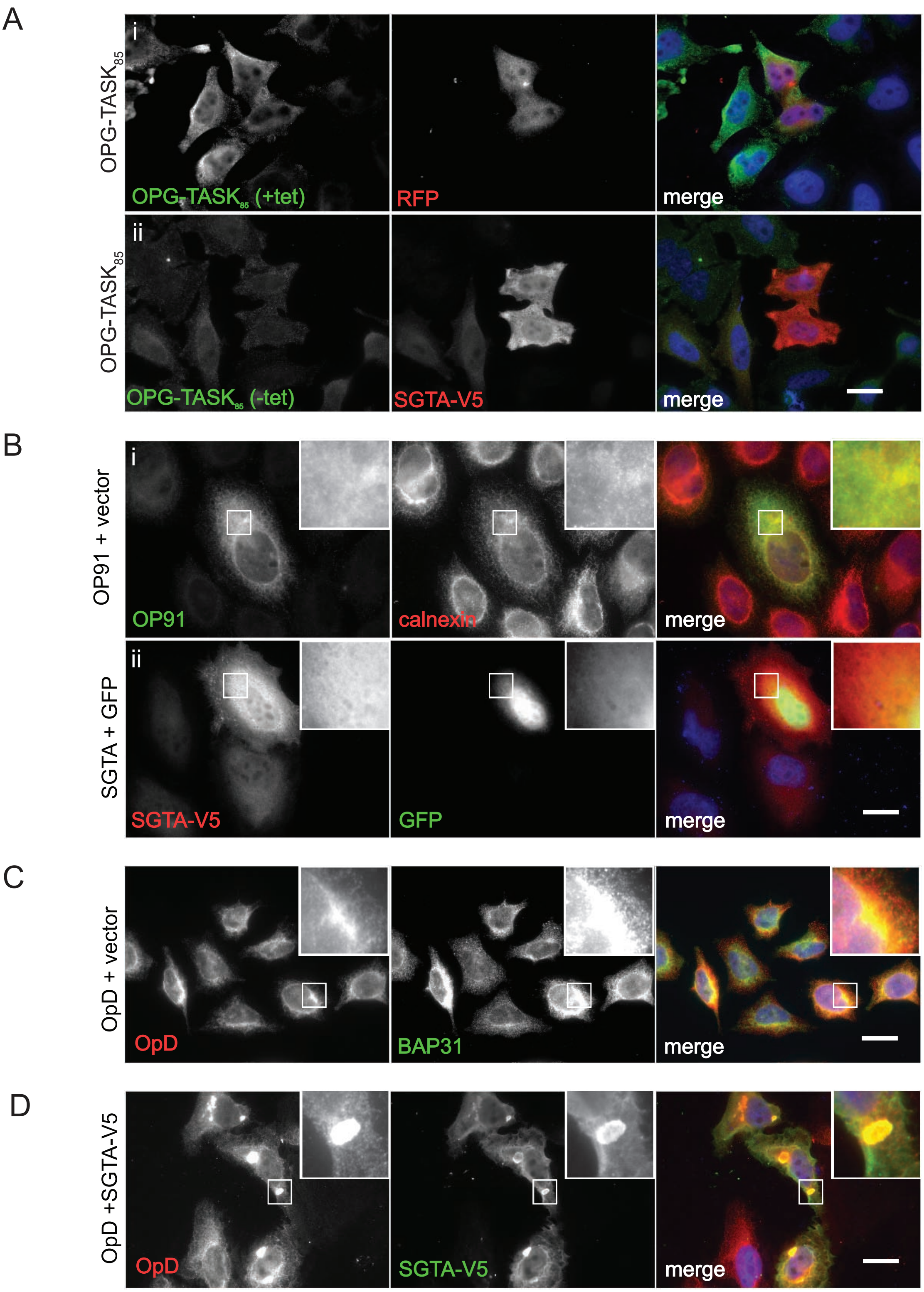
A
B
C
D
OPG-TASK85
i
OPG-TASK85 (+tet)
RFP
merge
ii
OPG-TASK85 (-tet)
SGTA-V5
merge
OP91 + vector
OP91
calnexin
merge
SGTA + GFP
SGTA-V5
GFP
merge
OpD + vector
OpD
BAP31
merge
OpD +SGTA-V5
OpD
SGTA-V5
merge

Figure S3

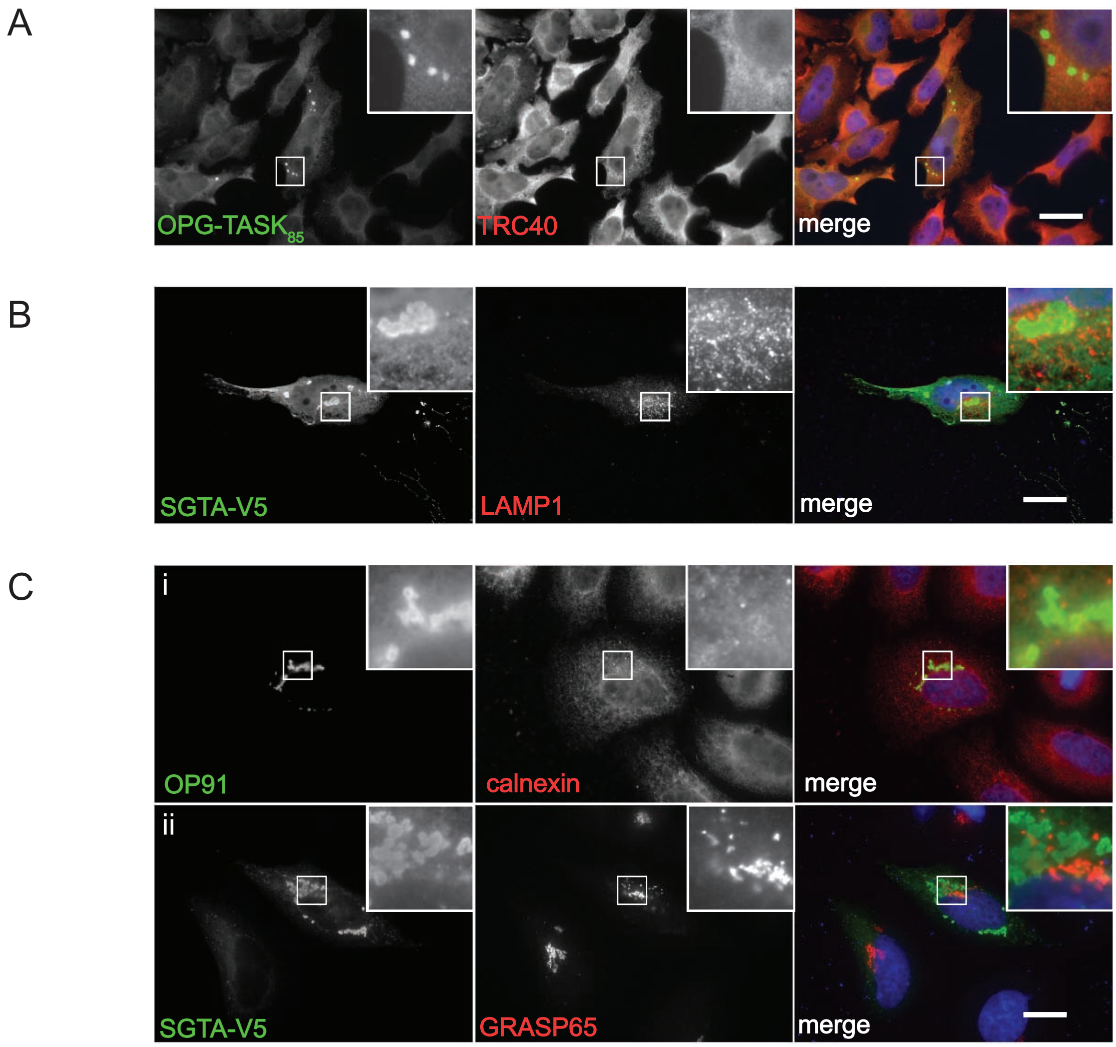
A
OPG-TASK85
TRC40
merge
B
SGTA-V5
LAMP1
merge
C
i
OP91
calnexin
merge
ii
SGTA-V5
GRASP65
merge

Figure S4
